# Functional network estimation using multigraph learning with application to brain maturation study

**DOI:** 10.1002/hbm.25410

**Published:** 2021-03-31

**Authors:** Junqi Wang, Li Xiao, Wenxing Hu, Gang Qu, Tony W. Wilson, Julia M. Stephen, Vince D. Calhoun, Yu‐Ping Wang

**Affiliations:** ^1^ Department of Biomedical Engineering Tulane University New Orleans Louisiana USA; ^2^ Department of Neurological Sciences University of Nebraska Medical Center, Omaha Nebraska USA; ^3^ Mind Research Network Albuquerque New Mexico USA; ^4^ Tri‐institutional Center for Translational Research in Neuroimaging and Data Science (TReNDS) Georgia State University, Georgia Institute of Technology, Emory University Atlanta Georgia USA

**Keywords:** brain maturation, functional connectivity, functional MRI, graph Fourier transform, Laplacian

## Abstract

Although most dramatic structural changes occur in the perinatal period, a growing body of evidences demonstrates that adolescence and early adulthood are also important for substantial neurodevelopment. We were thus motivated to explore brain development during puberty by evaluating functional connectivity network (FCN) differences between childhood and young adulthood using multi‐paradigm task‐based functional magnetic resonance imaging (fMRI) measurements. Different from conventional multigraph based FCN construction methods where the graph network was built independently for each modality/paradigm, we proposed a multigraph learning model in this work. It promises a better fitting to FCN construction by jointly estimating brain network from multi‐paradigm fMRI time series, which may share common graph structures. To investigate the hub regions of the brain, we further conducted graph Fourier transform (GFT) to divide the fMRI BOLD time series of a node within the brain network into a range of frequencies. Then we identified the hub regions characterizing brain maturity through eigen‐analysis of the low frequency components, which were believed to represent the organized structures shared by a large population. The proposed method was evaluated using both synthetic and real data, which demonstrated its effectiveness in extracting informative brain connectivity patterns. We detected 14 hub regions from the child group and 12 hub regions from the young adult group. We show the significance of these findings with a discussion of their functions and activation patterns as a function of age. In summary, our proposed method can extract brain connectivity network more accurately by considering the latent common structures between different fMRI paradigms, which are significant for both understanding brain development and recognizing population groups of different ages.

## Graphical abstract

We proposed a multigraph learning method to jointly estimate the brain connectivity networks from different paradigms. We further applied Graph Fourier transform to detect hub regions related to brain development in the low‐frequency components. Interesting findings have been made and discussed in a real‐world data set.

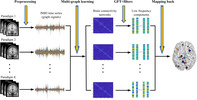

## 1 INTRODUCTION

Modern neuroimaging techniques provide an opportunity to study the human brain quantitatively. Especially, functional magnetic resonance imaging (fMRI) enables noninvasive measurements of both brain structure and its functional activities with a temporal resolution of seconds and with a spatial resolution of millimeters. It measures the low‐frequency fluctuations caused by hemodynamic response in blood‐oxygen‐level dependent (BOLD) signals and maps to the neural activities in the brain or spinal cord (Huettel, Song, & McCarthy, 2004), which facilitates the detection of correlations among distinct brain regions (Power et al., 2011). In particular, task‐based fMRI (tfMRI) analyses help to identify and characterize functionally distinct nodes in the human brain, providing the ability to interrogate the neural basis of mental functions and representations (Barch et al., 2013).

The human brain has attracted a significant number of studies due to its broad functional repertoire including action enabling, perception, and cognition. Despite a fixed anatomical structure, it has been a widely accepted assumption that the brain network architecture organizes local interactions to cope with diverse environmental demands (Park & Friston, 2013). Considerable research has been conducted to reveal the development of the brain from their functional activation differences throughout childhood and adolescence (Fair et al., 2009; Jolles, van Buchem, Crone, & Rombouts, 2010). In particular, task fMRI has been widely studied to explore task specific functional behavioral difference between child and adult brains (Passarotti et al., 2003), where different activation patterns were identified and reported for different age groups (Holland et al., 2001).

Recently, the graph signal processing (GSP) methodologies have become promising to tackle neuroimaging problems where observations are viewed as signals residing on the nodes of a graph (Hu et al., 2013; Sandryhaila & Moura, 2013; Shuman, Narang, Frossard, Ortega, & Vandergheynst, 2013). Within this framework, the brain is modeled as a graph or network consisting of a set of brain regions and pairwise relationships between these regions including connectivity or similarity measures. Spectral analysis was implemented to reveal the intrinsic structures of these graphs via the eigen‐analysis of their associated adjacency matrix, the graph Laplacian and their variants (Hu et al., 2013; Sandryhaila & Moura, 2013). A remarkable number of researches have been conducted to investigate the frequency domain behavior of the graph signals residing on these matrices (Chung, 1997), which facilitates the development of GSP (Shuman et al., 2013). Among them, the graph Fourier transform (GFT) has demonstrated its effectiveness in signal denoising (Shuman et al., 2013), the study of mental illness (Hu et al., 2013), and brain network analysis (Huang et al., 2016). Variants of GFT such as alternative graph Fourier transform (AGFT) (Sandryhaila & Moura, 2013) have been successfully applied to signal compression and label propagation. The rationality and advantages of these GSP tools over conventional Fourier transform have been illustrated by Shuman et al. (Shuman et al., 2013). These methods rely on a preselected kernel to construct the graph, presuming that the prior inner relationships among data samples are known. However, such information is unavailable because of the complexity of the brain with distinct task‐specific regions activated and deactivated among individuals. To overcome this issue, a data‐driven graph learning framework (Dong, Thanou, Frossard, & Vandergheynst, 2016) was proposed to better extract the graph structures. The authors further applied graph learning with graph Fourier transform and found applications in studying brain development (Wang et al., 2020).

It has been demonstrated that integrating multi‐modality or multi‐omics data can yield additional informative knowledge for the improvement of predictive accuracy (Kim et al., 2014; Sundermann, Herr, Schwindt, & Pfleiderer, 2014; Tsuda, Shin, & Schölkopf, 2005). In this work, we extend our model in (Wang et al., 2020) to incorporate multiple graphs, which can integrate multiple pieces of information to make diagnoses for patients. Specifically, we propose a multigraph learning framework to jointly learn the graph structure from multiple graphs. Afterward, we conduct GFT and design filters to detect essential brain regions in a specific frequency range to study the mechanisms of brain maturation. Finally, we apply the proposed model to neuroimaging data collected from the Philadelphia Neurodevelopmental Cohort (PNC) (Satterthwaite et al., 2014). It is a large‐scale collaborative study between the Brain Behavior Laboratory at the University of Pennsylvania and the Center for Applied Genomics at the Children's Hospital of Philadelphia, including healthy developing volunteers of age 8 to 22 years. The database contains 652 subjects with task fMRI observations available for both emotion identification and working memory tasks.

The contributions of this work can be summarized as follows.

• We propose a multigraph Laplacian learning framework to jointly estimate the graph structures from neuroimaging studies involving multiple paradigms. This is different from the previous graph integration methods (Kim et al., 2014; Tsuda et al., 2005), graph concatenated methods (Hu et al., 2013), and other multi task learning models (Wee, Yap, Zhang, Wang, & Shen, 2014), which combined the graphs estimated from single modality data linearly, calculated the graph from concatenated data, or estimated the identical graph among all the subjects, respectively. Instead, our model estimates multiple graphs with respect to different paradigms simultaneously by considering the overall brain network structures. The complementary information from multiple paradigms enables a more reliable construction of the brain network, resulting in improved detection of hub regions.

• We evaluate the proposed learning framework first on synthetic data, and then conduct extensive experiments on the real PNC data. The results demonstrate the superiority of the proposed method over traditional GSP approaches. A list of hub regions were identified revealing the different activation patterns in child and young adult group. A detailed discussion of the detected regions is given regarding their functions and relationships with brain maturation.

• We summarize our findings to demonstrate the consistency and discrepancy with previous studies and explore the potential biological patterns of the brain maturation process. Therefore, our work benefits the neuroimaging field by providing additional biomarkers related to brain development during adolescence.

The rest of the paper is organized as follows: in the Methods section, we provide a detailed description of signals defined on the graph and review the developments of applying GFT on these graph signals. We further propose a multigraph learning model to estimate the graph Laplacian matrix in the multi‐paradigm setting, followed by graph Fourier analysis and filter design. In the Results section, we validate the effectiveness of the proposed model on both synthetic and real data. Finally, we summarize the work in the Conclusion section.

## 2 METHODS

### 2.1 Signals defined on a graph

We use a graph to describe the pairwise relationships between a set of objects. For example, given a set of nodes *V* = *v*_*i*_, *i* = 1⋯*n*, a corresponding graph can be denoted as *G*(*V*, *W*), where *W* refers to a adjacency matrix of the relationships between each node‐pair. Brain functional connectivity aims to study the connection and activation pattern between different brain regions. As a result, we treat each brain region as a node and consequently the adjacency matrix *W* denotes the connections between different brain regions. A graph signal *x* ∈ ℝ^*n*^ is defined on the vertex set of the graph *G*, where the *i*‐th element of *x* represents the strength of the neural activity of the corresponding brain region.

We further introduce the graph Laplacian *L* defined below:

(1) L=D−W,

where *D* is the degree matrix, which is diagonal with the *i*‐th diagonal entry being the sum of the entries in the *i*‐th row of the weighted adjacency matrix *W*. The graph Laplacian *L* in (1) is real and symmetric, and thereby has a complete set of orthonormal eigenvectors {*f*_*i*_}_(*i* = 1,2,…,*n*)_. By eigendecomposition, we have *L* = *F*Λ*F*^*T*^, where Λ is the diagonal eigenvalue matrix Λ_*ii*_ = *λ*_*i*_, and *F* is the corresponding eigenvector matrix.

To further illustrate the behavior of the graph signal defined on the network, we define the smoothness (Laplacian regularization) of a graph signal *x* with respect to the Laplacian as (Belkin & Niyogi, 2003; Chan, Osher, & Shen, 2001; Shuman et al., 2013)

(2) $$S\left(x\right):=x^{T}\mathrm{Lx}=\frac{1}{2}\sum_{i,j=1}^{n}W_{\mathrm{ij}}{\left(x\left(i\right)-x\left(j\right)\right)}^{2},$$

where *x*(*i*) represents the element of *x* at *i*‐th node. It serves as an indicator reflecting the smoothness properties of a signal on the graph.

### 2.2 Related works

In this subsection, we review the recent developments in GSP especially graph Fourier transforms.

#### 2.2.1 GFT and AGFT

Suppose we have *m* ∈ ℕ subjects in total. For each subject, we have graph signals *X* = [*x*_1_, *x*_2_, …, *x*_*p*_] or [*x*^1^, *x*^2^, ⋯, *x*^*n*^]^*T*^ ∈ ℝ^*n* × *p*^ available, where *n* and *p* denote the number of nodes and graph signals. *x*_*i*_ ∈ ℝ^*n*^ is the *i*‐th column of *X* denoting the *i*‐th graph signal residing on the vertices, and *x*^*j*^ ∈ ℝ^*p*^ is the *j*‐th row of *X* denoting the observations of the *j*‐th vertex across the *p* graph signals. Let *W* = [*W*_*ij*_] ∈ ℝ^*n* × *n*^ denote the association or similarity matrix for a subject (Hu et al., 2013; Shuman et al., 2013).

(3) $$W_{\mathrm{ij}}=\left\{\begin{array}{ll} e^{-\frac{∥x^{i}-x^{j}{∥}_{2}^{2}}{2\sigma^{2}}} & \text{if}\;i∼j\\ 0 & \text{otherwise}, \end{array}\right.$$

where *i* ∼ *j* indicates an edge between node *i* and node *j*, and *σ* is the hyper‐parameter that determines the width of Gaussian distribution. The GFT of *x* with respect to *L* is defined as (Hu et al., 2013; Huang et al., 2016; Shuman et al., 2013)

(4) $$\tilde{x}:=F^{T}x.$$

The inverse GFT of $\tilde{x}$ with respect to *L* is defined as

(5) $$x=F\tilde{x}.$$

Note that *F* is orthonormal, that is, *F*^*T*^*F* = *I*, thus *x* and $\tilde{x}$ form a GFT pair.

The eigenvalues of the graph Laplacian carry the similar meaning as “frequency” to that of conventional Fourier transform. In fact, the eigenvalue reflects how much the corresponding eigenvector oscillates over the graph if we calculate the Laplacian regularization of the eigenvector *f*_*k*_:

(6) $$S\left(f_{k}\right):=f_{k}^{T}{\mathrm{Lf}}_{k}=f_{k}^{T}\lambda_{k}f_{k}=\lambda_{k}.$$

In (Sandryhaila & Moura, 2013, 2014), an alternative GFT was proposed to expand graph signals with respect to the eigenfunctions of the corresponding adjacency matrix. Suppose the adjacency matrix *A* is available and its Jordan decomposition *A* = *VJV*^−1^, where *J* is the Jordan normal form and *V* is the matrix of generalized eigenvectors. The alternative GFT and its inverse of a given graph signal *x* can be defined with respect to *A* as in (4), (5).

#### 2.2.2 Graph Laplacian learning based Fourier transform (GLFT)

In order to avoid the deviation of pre‐selected kernels in constructing the network, the authors in (Dong et al., 2016) proposed a data‐driven learning framework to better estimate the topology of the graph.

Suppose we have graph signals *X* = [*x*_1_, *x*_2_, ⋯, *x*_*p*_] ∈ ℝ^*n* × *p*^ observed on a graph with *n* nodes. The smooth signal *Y* and the graph Laplacian were estimated by minimizing the Laplacian regularization term as well the loss *Y* (Dong et al., 2016), where Frobenius norm penalty of *L* and constrains were applied to ensure the learned graph Laplacian is nontrival and valid. Detailed description can be found in File S1.

Notably, the proposed learning algorithm was further combined with GFT in (Wang et al., 2020) to split the graph signals into different frequency components. Given a graph signal *x* and its learned graph Laplacian, the GLFT and the inverse of *x* can be defined with respect to *L* as in (4), (5).

### 2.3 Multigraph analysis

#### 2.3.1 Multigraph Laplacian learning

Graph signals from different views or paradigms may provide complementary information for better characterizing the network structure. In this section, we extend the learning framework into multiple graphs setting. To begin, we introduce the notions of the multigraph signals. For a subject with graph signals defined on *d* modalities, we use $\left[x_{1}^{\left(k\right)},x_{2}^{\left(k\right)},\ldots ,x_{p_{k}}^{\left(k\right)}\right]=X^{\left(k\right)}\in {\mathbb{R}}^{n\times p_{k}}$ to represent the set of graph signals from the *k*‐th(*k* = 1, 2, …, *d*) modality, where *p*_*k*_ is the number of graph signals from the *k*‐th modality. For each modality, by taking the smoothness into consideration, the graph Laplacian can be easily estimated (File S2).

In multigraph scenario, we still adhere to the assumption that the graph signals have a small total variation on the estimated graph for each modality. This assumption reflects a better fit between the learned graph structure and the graph residing on it. Moreover, since the multiple signals come from the same subject, common structures reflecting interrelationships between modalities should exist. Based on these assumptions, it is natural to generalize the graph Laplacian learning framework into a multigraph setting using the following objective function.

(7) $$\begin{array}{l} \min_{L^{\left(1\right)},\ldots ,L^{\left(d\right)}}\;\frac{1}{d}\sum_{k=1}^{d}\left[\mathrm{tr}\left(X^{\left(k\right)T}L^{\left(k\right)}X^{\left(k\right)}\right)+\beta ∥L^{\left(k\right)}{∥}_{F}^{2}\right]\\ +\sum_{i,j=1}^{d}\alpha_{\mathrm{ij}}∥L^{\left(i\right)}-L^{\left(j\right)}{∥}_{F}^{2}\\ s.t\;\mathrm{tr}\left(L^{\left(k\right)}\right)=n,\\ \begin{array}{l} L_{\mathrm{ij}}^{\left(k\right)}=L_{\mathrm{ji}}^{\left(k\right)}\leq 0,\left(i\neq j\right),\\ L^{\left(k\right)}\cdot 1=0,\left(k=1,2,\ldots ,d\right). \end{array} \end{array}$$

Compared with the single variate learning framework (File S2), we considered the overall graph structures by regulating their difference among modalities. The positive parameters *α*_*ij*_ are the key factors reflecting the latent structure shared by different views or modalities. If *α*_*ij*_ is set to 0, which means that the graph structures are considered independently with each other, the solutions are equivalent to learning the graph structure from each modality separately as in (File S2). If *α*_*ij*_ is set to infinity, which forces the graph structures to be the same among modalities, the solutions are equivalent to learning the graph with the concatenated method. Ideally, *α*_*ij*_ should be set as the degree of similarity measurement between modalities with certain prior information. However, in our case, the inner‐relation between modalities is rather sophisticated, so *α*_*ij*_ is tuned through cross‐validation in this work. *β* is a positive regulator that controls the distribution of the learned graphs and we tune it through cross‐validation as well. Additional constraints are enforced to prevent the null solutions and to ensure that the learned Laplacian matrices are valid. The optimization problem can be effectively solved with CVX toolbox (Grant, Boyd, & Ye, 2015).

#### 2.3.2 Graph Fourier transform

After obtaining graph Laplacian *L*^(*k*)^ for each modality, we are able to conduct GFT of *x*^(*k*)^ in each modality with respect to *L*^(*k*)^ as

(8) $${\tilde{x}}^{\left(k\right)}:=F^{\left(k\right)T}x^{\left(k\right)}.$$

where *F*^(*k*)^ is the eigenvector matrix of *L*^(*k*)^. In addition, the inverse GFT of ${\tilde{x}}^{\left(k\right)}$ with respect to *L*^(*k*)^ is defined as

(9) $$x^{\left(k\right)}=F^{\left(k\right)}{\tilde{x}}^{\left(k\right)}.$$

As *F*^(*k*)^ is orthonormal, the original signal *x*^(*k*)^ can be recovered through the inverse GFT of ${\tilde{x}}^{\left(k\right)}$. Thus, *x*^(*k*)^ and ${\tilde{x}}^{\left(k\right)}$ form a GFT pair.

Remarkably, the GFT can be reduced to conventional Fourier transform for specific graphs such as a cycle graph (Huang et al., 2016), where the eigenvectors in *F* are expressed as $f_{k}={\left[1,e^{-i\frac{2\pi \left(k-1\right)}{n}},\ldots ,e^{-i\frac{2\pi \left(k-1\right)\left(n-1\right)}{n}}\right]}^{T}$. On the other hand, as defined in (6), the eigenvalue reflects the smoothness of the corresponding eigenvector over the graph. Thus, the GFT naturally divides the original graph signal into different “frequency” components: small eigenvalues oscillate slowly, appearing to be smooth, whereas the large eigenvalues account for high frequencies, and the associated eigenfunctions oscillate rapidly.

#### 2.3.3 Graph filtering

As discussed above, the transformed signals can be manipulated into a range of frequencies to facilitate extracting information and exploring their behaviors. Following the standard procedure from signal processing, we design a low‐pass filter, a high‐pass filter, and a band‐pass filter based on eigenvalues to split the frequency components (see supplementary section 3).

Based on the designed filters, the graph signals were naturally divided into low, intermediate, and high frequency components as $\tilde{x}={\tilde{x}}_{\mathrm{low}}+{\tilde{x}}_{\mathrm{mid}}+{\tilde{x}}_{\text{high}}$. Accordingly, the original graph signals can be recovered by *x* = *x*_*low*_ + *x*_*mid*_ + *x*_*high*_ with *x*_*low*_, *x*_*mid*_, *x*_*high*_ being the reconstructed signals from the respective frequencies.

Brain network theory has demonstrated the co‐existence of disorganized behavior and ordered regularity in the brain functional network (Sporns, 2010). The organized part reveals the functional or structural information of the brain network in a large population, such as the default mode network (DMN) (Buckner, Andrews‐Hanna, & Schacter, 2008), sensory motor network (Gallese & Lakoff, 2005), while the disorganized part reflects the individual differences among subjects. In our multigraph learning framework, the ordered part corresponds to low frequency components that are smooth across the graph representing the main contribution of the brain functional operations, while the disordered portion accounts for the high frequency component with a higher chance of capturing noise or individual differences. The low variability has proven to be essential in the analysis of neurological disease and behavior and the learning process (Garrett, Kovacevic, McIntosh, & Grady, 2012; Huang et al., 2016). As the main motivation of this work is to analyze the brain functional connectivity patterns at different pubertal stages, we put our emphasis on the learning of the low frequency subspace in the following section.

## 3 RESULTS

**FIGURE 1:**
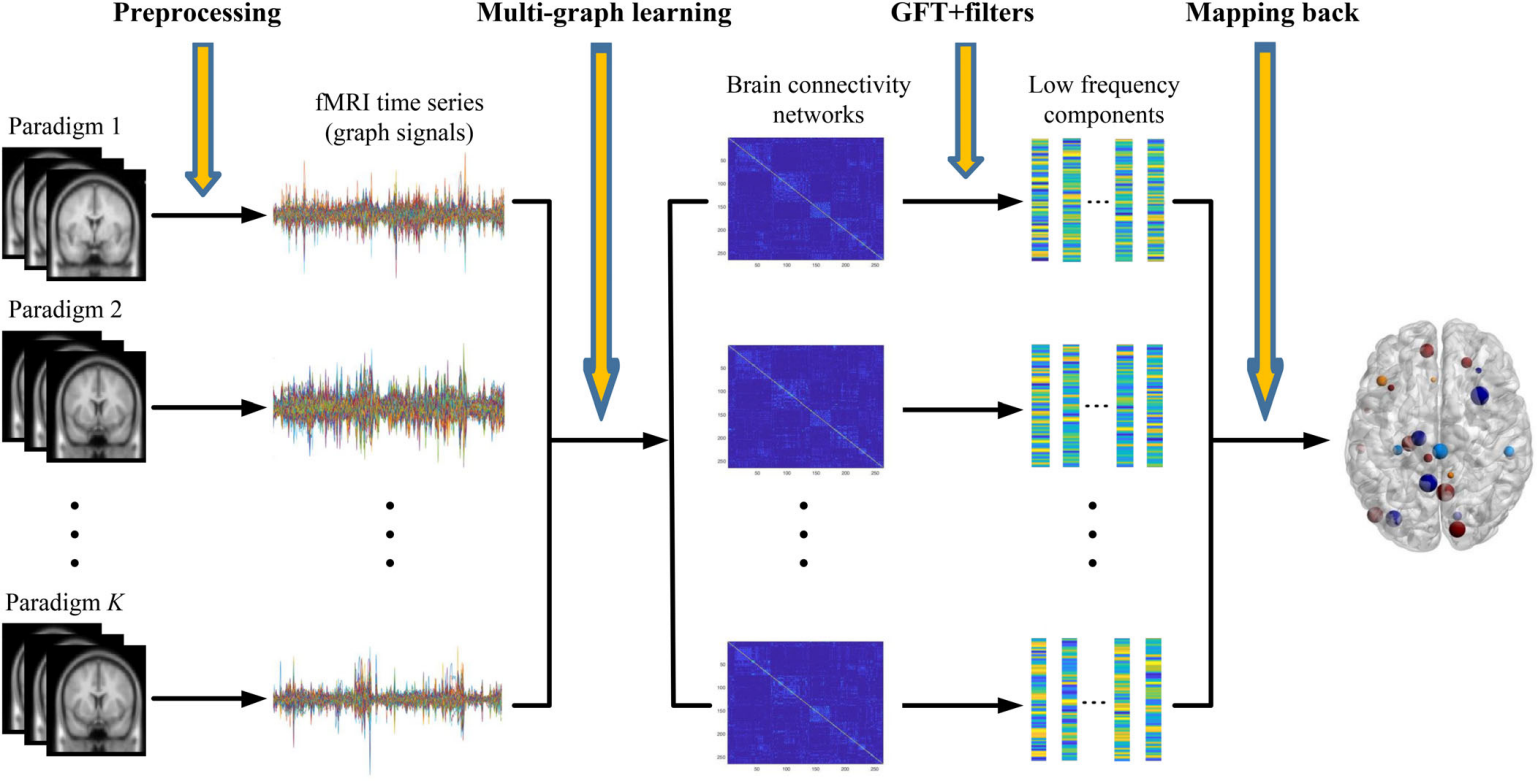
A flow diagram of multigraph signal analysis of one subject for brain network construction and hub regions identification. There are four major steps involved. In the preprocessing step: task fMRI measurements from multi‐paradigm were preprocessed to become graph signals defined on the parcellated brain regions according to the Power atlas (Power et al., 2011). In the multigraph learning step: we applied the proposed multigraph Laplacian learning algorithm to construct the brain connectivity networks simultaneously for each paradigm. In the GFT‐filters step: we conducted graph Fourier transform and designed proper filters to transform the original signals into the frequency domain and put our emphasis on the low frequency components. Finally, in the mapping back step: we analyzed the energy distribution of the eigenfunctions and mapped these back to their corresponding brain regions, where regions with significant contribution were identified

We evaluated the performance of our proposed multigraph Fourier transform framework using both synthetic and real data analysis in this section. The comparison between other graph Fourier transform related methods such as GFT, alternative GFT, and the graph learning based Fourier transform was demonstrated (Sandryhaila & Moura, 2013; Shuman et al., 2013; Wang et al., 2020). Meanwhile, hub regions related to brain maturation were identified from task‐related fMRI, followed by a significant test of the detected regions. Finally, we calculated the consistency between subjects for each group and conducted a classification test to further validate the performance of our proposed method. In short, our proposed multigraph learning method enabled a more precise characterization of the brain FCN with multi‐paradigm fMRIs, and thus facilitates the study of brain development. For clarification, we illustrated the major steps involved in real data analysis in Figure 1.

### 3.1 Synthetic data analysis

#### 3.1.1 Artificial graphs construction

To evaluate the performance of our proposed model, we conducted an experiment on synthetic multigraph data. Specifically, we synthesized artificial graphs with 20 vertices following the Erdos‐Renyi (ER) model (Erdos & Rényi, 1960) and the Barabasi‐Albert (BA) model (Barabási & Albert, 1999). The ER model first generates the whole node set, and then each edge randomly with a .2 probability. The BA model, by contrast, adds nodes and edges one by one, according to the degree distribution of the existing nodes, based on the assumption that there is a higher chance for a new vertex connecting to an existing node with higher degree. The ground truth graph Laplacian was obtained from (1), where the edges were binarized. For each artificial graph, a total of 100 signals were yielded following a zero‐mean multivariate Gaussian distribution as follows

(10) $$x∼\;N\left(0,F\Lambda^{†}F^{T}+{\sigma_{ɛ}}^{2}I_{n}\right)).$$

We set $\sigma_{ɛ}^{1}=0.3$ and $\sigma_{ɛ}^{2}=0.5$ for two paradigms, respectively.

#### 3.1.2 Result and comparison

Given the synthesized multi‐paradigm signals on each graph, we first applied the proposed learning framework to construct the graph Laplacian matrix. For comparison, we also calculated the graph Laplacian based on single‐paradigm data, which demonstrated superiority over GFT and AGFT (Wang et al., 2020). For ease of comparison, the learned graph Laplacian matrices were binarized at a threshold 10^−4^. Precision, recall, and F‐measure were adopted as the criteria to evaluate the performance of these methods statistically. We displayed the averaged results and their *SD* from 20 repeated experiments in Table 1. The results demonstrated that the constructed graph networks using the multigraph learning framework outperformed those using the single‐paradigm based method. In other words, the overall structural information from multiple paradigms offers advantages in better constructing the graph than in single paradigm. The comparatively poorer performance of the ER model was most likely caused by the higher randomness against smoothness condition.

**TABLE 1:** Simulation results

BA model			
Graphs	F‐measure	Precision	Recall
2D‐L1	0.9870 ± 0.0215	0.9474 ± 0.0292	0.9921 ± 0.0193
2D‐L2	0.9936 ± 0.0114	0.9875 ± 0.0222	1.0000 ± 0.0000
L1	0.8723 ± 0.0954	0.8539 ± 0.0920	0.8921 ± 0.1017
L2	0.8545 ± 0.0815	0.8366 ± 0.0801	0.8737 ± 0.0861
**ER model**			
Graphs	F‐measure	Precision	Recall
2D‐L1	0.7892 ± 0.0337	0.7848 ± 0.0335	0.7937 ± 0.0344
2D‐L2	0.8033 ± 0.0240	0.7984 ± 0.0248	0.8083 ± 0.0240
L1	0.7043 ± 0.0288	0.6993 ± 0.0300	0.7094 ± 0.0283
L2	0.7380 ± 0.0272	0.7324 ± 0.0273	0.7438 ± 0.0280

*Note:* 2D‐L1 and 2D‐L2 represent the graphs jointly estimated from two paradigm signals. L1 and L2 are the graphs recovered from graph learning algorithm for single paradigm.

**FIGURE 2:**
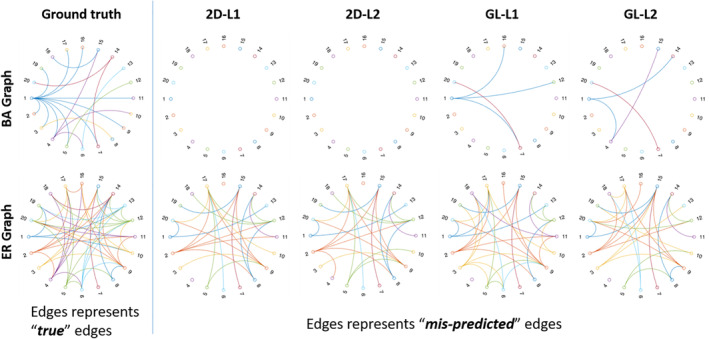
The ground truth graphs versus the reconstructed graphs from our proposed multigraph learning framework and the graph learning method for single view data. Note that the edges in the last four sub figures represent the falsely predicted edges. The upper part is the simulation from BA model and the lower part is the ER model. 2D‐L1 and 2D‐L2 represent the Laplacian matrices estimated from the proposed multi‐paradigm learning framework. GL‐L1 and GL‐L2 represent the Laplacian matrices learned from single view graph signals

For clarity, we also visualized the learned graph Laplacian matrices with respect to both BA and ER graphs in Figure 2. The experiments are repeated 20 times and the visualization is based on the results from the last time. Overall, our proposed method gives a better reconstruction of the ground truth graph compared with the single view based method.

### 3.2 Real data analysis

We further applied the proposed model to fMRI observations collected from the Philadelphia Neurodevelopmental Cohort (PNC) (Satterthwaite et al., 2014) with a focus on the brain maturation process. MRI examinations were conducted on a single 3 T Siemens TIM Trio whole‐body scanner. The images were collected using a single‐shot, interleaved multi‐slice, gradient‐echo, echo planar imaging sequence.

#### 3.2.1 Data acquisition and preprocessing

In this study, we consider two types of task MRI data from PNC (Satterthwaite et al., 2016) to reveal the brain network difference between child and young adult given the same cognitive task. The two tasks involve emotion identification and working memory examination, and a detailed description of the experiments can be found in the paper by Satterthwaite et al. (2014). For each subject, both task based fMRI time series were available and parcellated based on the same atlas.

The emotion identification task employs a fast event‐related design with a jittered inter‐stimulus interval (ISI). Participants were required to label the emotions displayed by the trained actors (50% female) with neutral, happy, sad, angry, or fearful expressions. There were 60 faces in total in color photographs that were rated and selected by professional directors. Each face was displayed for 5.5 s followed by a variable ISI from 0.5 to 18.5 s, during which a complex crosshair (that matched the faces' perceptual qualities) was displayed. The task duration was 10.5 min (Satterthwaite et al., 2014).

The working memory task involved a fractal version of the standard n‐back task, which has proved to be a reliable probe of the executive system and was beneficial to avoid lexical processing abilities that evolve during development (Brown et al., 2004; Schlaggar et al., 2002). The task involved presentation of complex geometric figures (fractals) for 500 ms, followed by a fixed ISI of 2,500 ms. There are three levels of working memory load in total: 0‐back, 1‐back, and 2‐back. In 0‐back setting, participants responded to a specified target fractal; in 1‐back setting, participants responded if the current fractal was identical to the previous one; in 2‐back setting, participants responded if the current fractal was identical to the previous two trails. The task duration was 11.6 min (Satterthwaite et al., 2014).

We followed a standard pipeline to preprocess the data using SPM12 (Ashburner et al., 2014), consisting of motion correction, spatial normalization to standard MNI space (adult template) and spatial smoothing with a 3 mm FWHM Gaussian kernel. We applied regression to remove the effect of motion (6 parameters), followed by a band‐pass filter in 0.01 Hz to 0.1 Hz frequency range. Next, we further mapped the imaging data into 264 regions of interest based on the Power atlas (Power et al., 2011) with a sphere radius 5 mm. Finally, we extracted the ROI‐level fMRI time series by averaging the time sequences of all voxels in the same ROI to get a 264 × *p* matrix for each subject with *p* = 210 and *p* = 231 denoting the number of time points for emotion identification and working memory tasks, respectively, due to the total duration of the MRI scan.

Since we focus on the brain development analysis as a function of age, we subdivide the subjects into child and young adult groups. Specifically, subjects whose age was below 12 years were considered to belong to the child group, while subjects over 18 years belong to the young adult group, as shown in Table 2. Chi‐Square statistic test (McHugh, 2013) was performed and there was no significant difference in distribution of males and females between the child and young adult groups (*p* = .2878).

**TABLE 2:** Characteristics of the subjects in this study

Group	Age (Mean ± *SD*)	Male/female
Child	10.2926 ± 0.9358	61/74
Young adult	19.2606 ± 0.9952	57/92

#### 3.2.2 Parameter tuning/ effect of parameters

We illustrated the determination of the important parameters involved in the proposed framework, including *α* that controls the similarity between paradigms, *β* that regulates the off diagonal entrees of the graph Laplacian, and *l* that determines the cutoff of the low frequency components. The parameters were determined separately and 10‐fold cross validation was applied to evaluate the performance of the parameters. Specifically, *β* was initially set in the range of [10^−5^, 10^−4^, …, 10^4^, 10^5^], *α* was initially set in the range of [10^−2^, 10^−1^,1,10,100] × *β*, and cutoff *l* was set in [20, 60]. The cross validation criteria was the classification accuracy of the estimated graph Laplacian towards their corresponding age groups.

**FIGURE 3:**
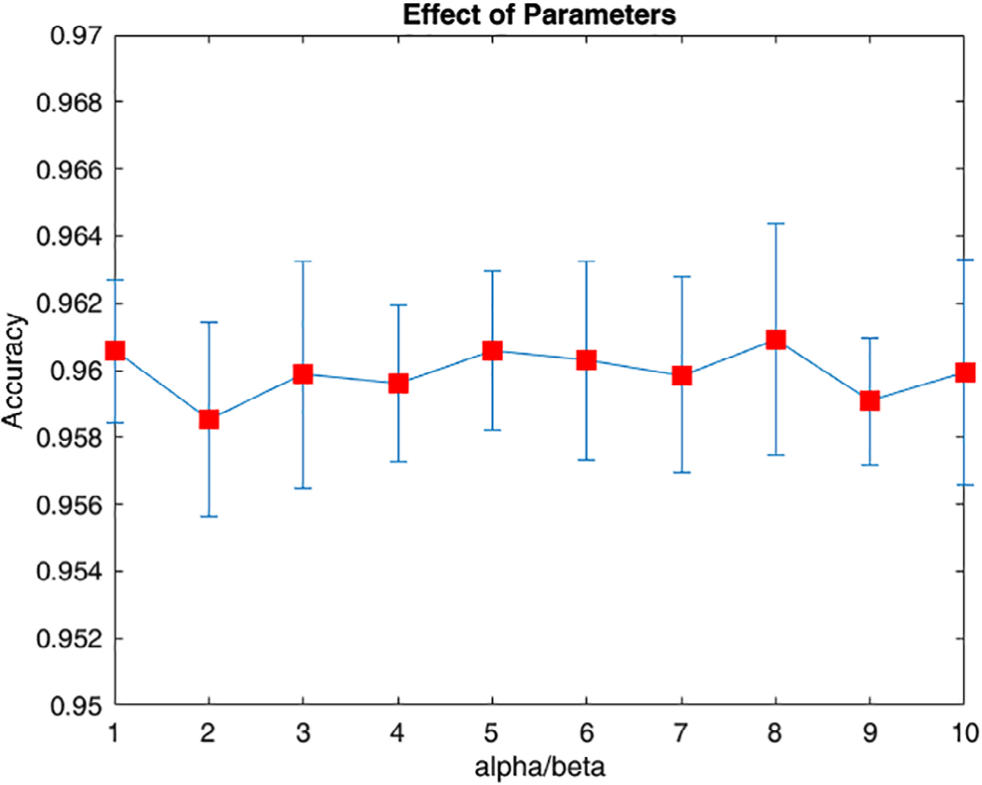
The visualization of the sets of parameters *α*, *β* versus their corresponding classification accuracy in classifying different age groups. The *β* is set to 10^3^ and the x‐axis is the *α*/*β*. For each pair of the parameters, the classification task was repeated 10 times and the averaged cross‐validated accuracy and standard derivation were displayed. We concluded that the classification performance is solid and robust (over 95% accuracy and fluctuation is within 3 × 10^−3^) with a proper range of the parameters. A similar result is observed when *β* is at a proper range, which demonstrated the robustness of our model

Based on the objective function (7), a large *β* (over 10^4^) leads to a uniform distribution of the learned graph Laplacian (the diagonal close to one and the off‐diagonal tends to be the same) and a small *β* (below 1, compared with the scale of the Laplacian regularization term controlling the smoothness) leads to an extremely sparse solution where a clear majority of off‐diagonal elements equal 0. The parameter *α* reflects the inner structural relationships between paradigms by controlling the shared latent information. We visualized the relationship of the parameters (*α*/*β*) versus cross‐validation accuracy to evaluate the effect of the parameters in Figure 3. The cutoff splitting the frequency was also selected via cross‐validation, where 10‐fold cross validation was performed on the train set with *l* in [20, 60]. We finally adopted *l* = 48, and *α*/*β* = 7 as the low frequency boundary and parameter for the optimization algorithm. In fact, the classification performance was robust across a range of both parameters.

#### 3.2.3 Detection and visualization of hub regions

**FIGURE 4:**
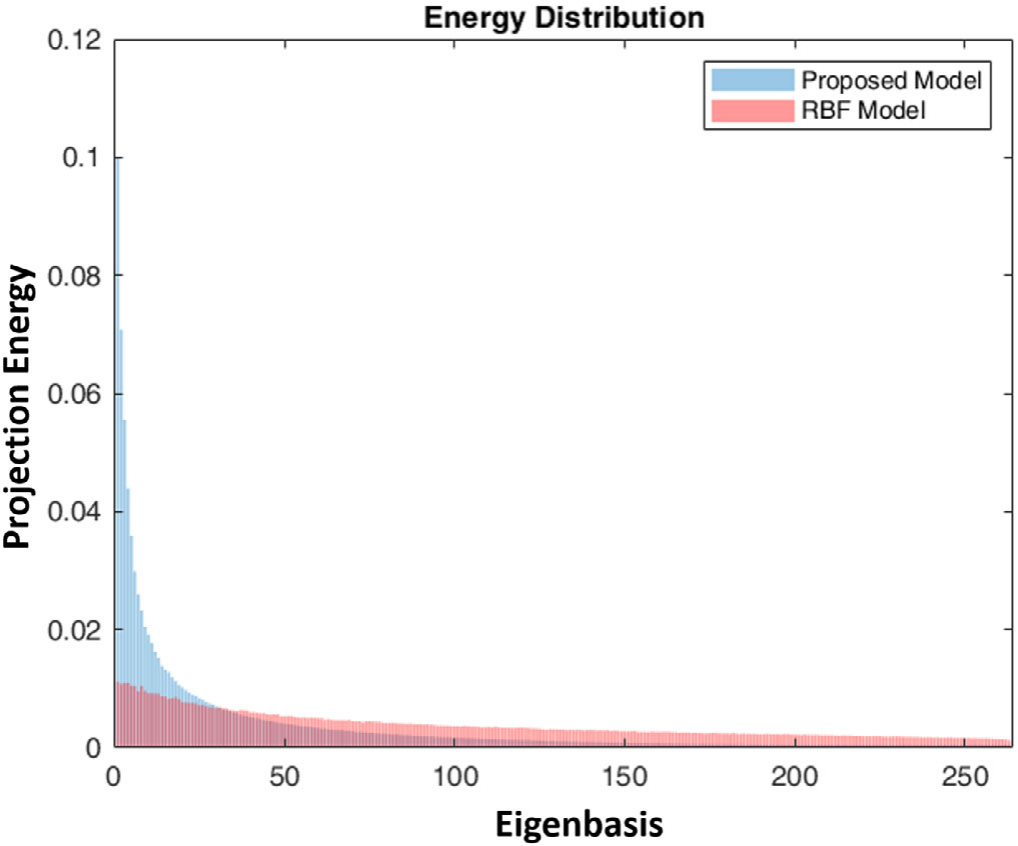
The comparison of projection energy between our proposed method and the RBF (Radial basis function) kernel based method involved in the traditional graph Fourier transform (Shuman et al., 2013). The projection energy was calculated based on the averaged projection power across all the subjects and normalized to have unified area under the curve. The eigenbases are sorted in an increasing manner with corresponding eigenvalues except for the first one, which has constant value in its eigenfunction. Our proposed method enforced the extraction of the features in the low frequency components compared with the traditional methods

**FIGURE 5:**
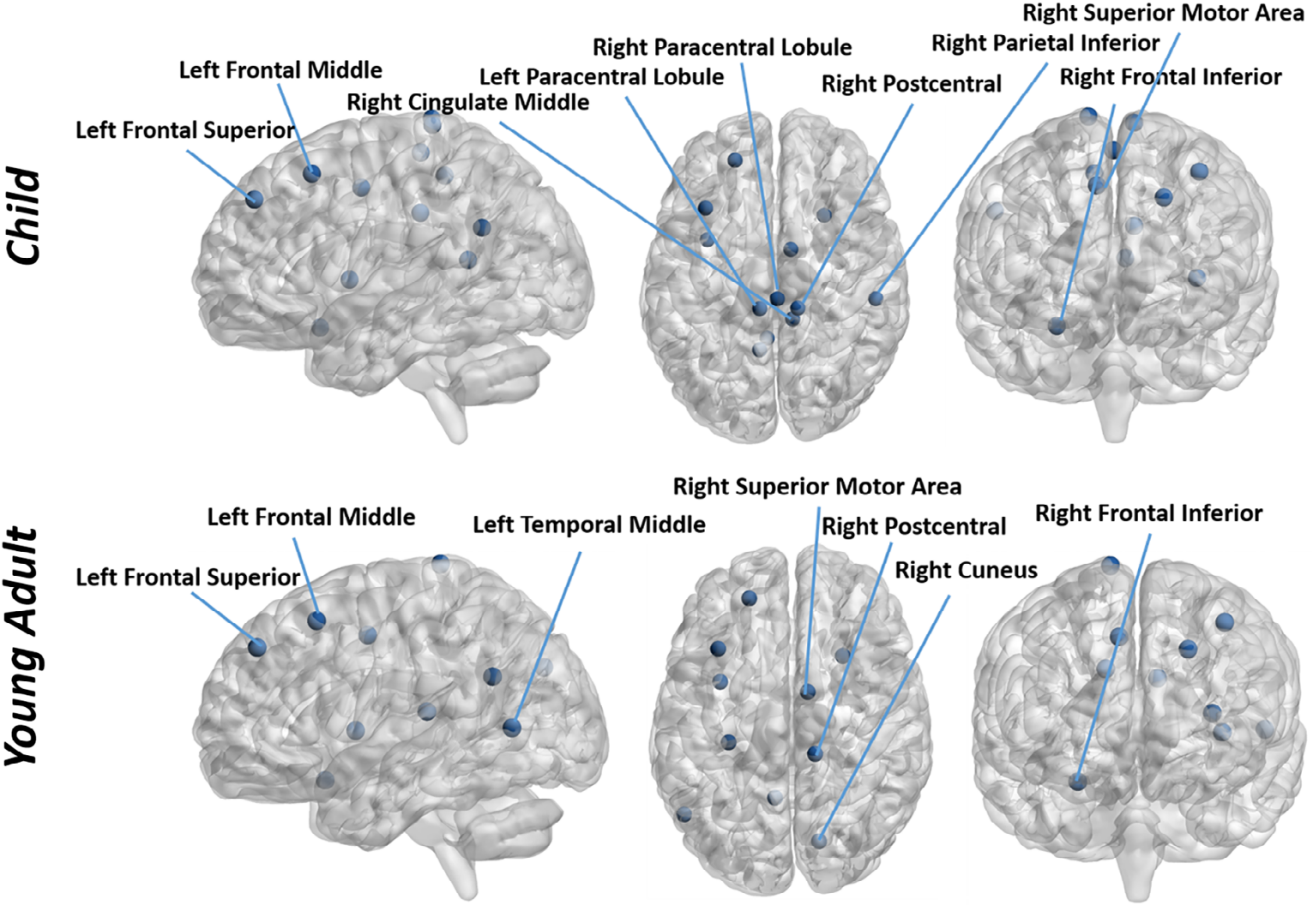
The visualization (sagittal, axial, and coronal views) of the detected hub regions from task fMRI analysis. The regions in the upper part of the figure are identified from the child group while the regions in the lower part of the figure are detected from the young adult group. The visualization of the brain network is by BrainNet Viewer (Xia, Wang, & He, 2013)

As illustrated in Figure 1, we applied our proposed framework to detect age related biomarkers using imaging data from two task‐based fMRI paradigms. Specifically, fMRI observations at each time point were treated as graph signals defined on the parcellated brain regions. Multigraph Laplacian learning algorithm was applied on both working memory and emotion identification tasks to estimate the graph structure for each subject. We conducted graph Fourier transform and designed filters afterwards to divide the graph signals into low, intermediate and high frequency components. Based on the explanations at the end of method section, we put our emphasis on the low frequency portion. In order to identify hub regions for each age group, we calculated the projection energy on eigenbasis in the low frequency range, where the spikes of the energy concentration were selected and further mapped back to their corresponding cortical regions. Specifically, the inner products of the transformed signals in the low frequency domain on each eigenbasis were computed and two standard deviations higher than mean value (Bassett et al., 2008) was adopted as the threshold to select hub regions. The energy distribution of the transformed signals provided additional evidence on the superiority of our proposed method (Figure 4). The relatively higher energy concentration over competing method in the low frequency ranges indicated that our proposed method could facilitate the extraction of the information in the organized brain regions, which in turn improved the subsequent hub identification and classification. We investigated the group differences of the detected hub regions using standard two‐sample *t* test to estimate their significance. We summarize the results in Table 3, where previous reported regions are marked with *. Detailed illustrations of these regions and their literature references are given in Discussion Section. For ease of visualization, we display the locations of the identified hub regions related to brain maturity in Figure 5 with the help of BrainNet Viewer (Xia et al., 2013).

**TABLE 3:** Identified hub regions in different age groups

	Child		Young adult	
Common regions	Location	*p*‐value	Location	*p*‐value
Left cingulate gyrus*	2.6606*e*^−4^	Left cingulate gyrus*	2.6606*e*^−4^	
Right cingulate gyrus*	1.8612*e*^−10^	Right cingulate gyrus*	1.8612*e*^−10^	
Right postcentral gyrus*	.0439	Right postcentral gyrus*	.0439	
Right medial frontal gyrus *	2.3261*e*^−6^	Right medial frontal gyrus *	2.3261*e*^−6^	
Left posterior cingulate*	2.1909*e*^−4^	Left posterior cingulate*	2.1909*e*^−4^	
Right inferior parietal lobule*	.0019	Right inferior parietal lobule*	.0019	
Left Claustrum	4.4006*e*^−4^	Left Claustrum	4.4006*e*^−4^	
Left superior frontal gyrus*	1.3694*e*^−5^	Left superior frontal gyrus*	1.3694*e*^−5^	
Right inferior frontal gyrus*	.0035	Right inferior frontal gyrus*	.0035	
Left middle frontal gyrus*	7.7311*e*^−4^	Left middle frontal gyrus*	7.7311*e*^−4^	
	Left middle temporal gyrus*	2.3678*e*^−4^	Left Postcentral gyrus	.0796
Different regions	Right cuneus*	.1352	Right paracentral lobule*	8.9501*e*^−5^
	Left insula	.0072		
	Right inferior temporal gyrus*	4.73*e*^−10^		

*Note:* Previously reported regions are marked with *.

#### 3.2.4 Subject consistency

**FIGURE 6:**
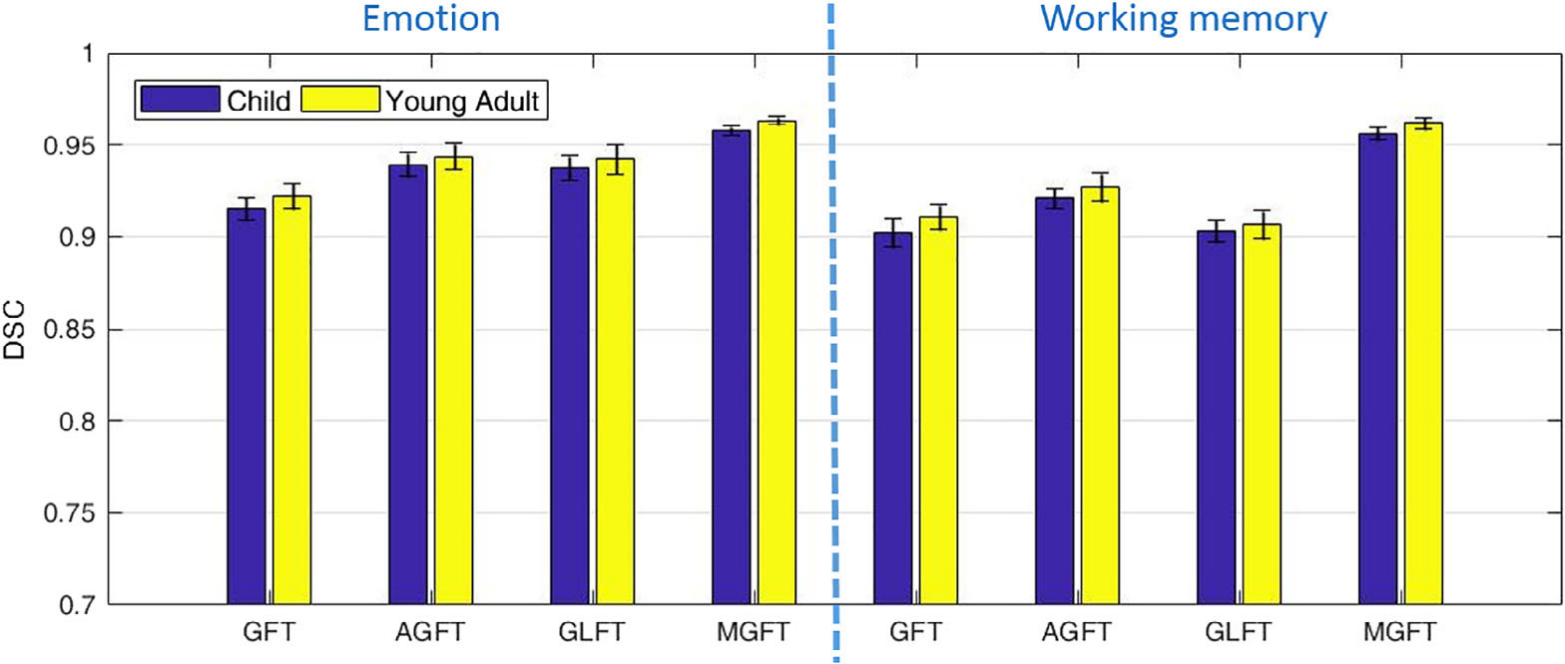
Dice similarity coefficient (DSC) values for the proposed methods and the competing methods

To validate our previous statement that organized brain functional and structural information should be consistent within a large population, the subject consistency can be adopted as an indicator to compare the subjects' behavior in the same group (Li et al., 2019). It is well known that Dice similarity coefficient (DSC) is a commonly used metric to quantify the subject consistency:

(11) $$\mathrm{DSC}=\frac{2\mathrm{TP}}{2\mathrm{TP}+\mathrm{FP}+\mathrm{FN}},$$

where *TP*, *FP*, and *FN* denote the true positive, false positive, and false negative, respectively. A high DSC score indicates a high consistency of the functional connectivity networks among subjects from the same population. We calculated the DSC values based on our proposed method and the competing methods for child and young adult group, respectively, shown in Figure 6. The DSC scores of our proposed method were higher than the DSC sores of all the comparison methods, demonstrating an increase of the subject consistency in the learned brain connectivity networks. Furthermore, the DSC values of the young adult group were always higher than those of the child group, suggesting an increase of consistency with increased age. In other words, the brain functional network continues to change and stabilize through adolescence, which is in line with the previous studies and common assumptions of brain development (Arain et al., 2013; Fair et al., 2009; Jolles et al., 2010).

#### 3.2.5 Classification

To further illustrate our findings, we conducted classification experiments to separate different age groups with the learned brain network. Specifically, we considered the connectivity matrix jointly estimated for each subject as the input to a linear support vector machine (SVM) classifier (Schölkopf & Smola, 2002) to divide subjects into child or young adult groups. We also performed classification tasks with the constructed networks using graph Laplacian learning based Fourier transform, GFT and alternative GFT for comparison (Sandryhaila & Moura, 2013; Shuman et al., 2013; Wang et al., 2020).Sensitivity, specificity and accuracy were adopted as the criteria to evaluate the performance. We repeated the prediction experiments 10 times with 10‐fold cross‐validation to avoid occasionality and overfitting and displayed the result in Table 4.

**TABLE 4:** Classification Results for fMRI measurements

emoid‐fMRI			
Method	ACC	SEN	SPE
MGFT	0.9606 ± 0.0022	0.9385 ± 0.0036	0.9805 ± 0.0021
GLFT	0.9401 ± 0.0080	0.9448 ± 0.0106	0.9349 ± 0.0085
GFT	0.9187 ± 0.0047	0.9230 ± 0.0078	0.9140 ± 0.0087
AGFT	0.9415 ± 0.0033	0.9491 ± 0.0064	0.9332 ± 0.0073
nback‐fMRI			
Method	ACC	SEN	SPE
MGFT	0.9592 ± 0.0034	0.9378 ± 0.0038	0.9785 ± 0.0053
GLFT	0.9050 ± 0.0106	0.9331 ± 0.0071	0.8743 ± 0.0187
GFT	0.9066 ± 0.0079	0.9069 ± 0.0105	0.9067 ± 0.0104
AGFT	0.9237 ± 0.0057	0.9309 ± 0.0049	0.9164 ± 0.0107

*Note:* ACC, SEN, and SPE are the abbreviations of accuracy, sensitivity, and specificity, respectively. We display the result for mean value ± *SD*. MGFT, GLFT, and AGFT is the short for multi GFT analysis, graph Laplacian learning based Fourier transform and alternative GFT, respectively.

In summary, the proposed multigraph learning method outperformed the other GFT GSP methods. Our approach was able to extract useful shared latent information from multi‐paradigms, resulting in improved classification performance, which increased the reliability of the detected regions associated with brain maturation during puberty.

## 4 DISCUSSION

### 4.1 The most discriminative regions

In this section, we provided a detailed investigation of the most discriminative regions selected by our method to explore their relationships with brain maturation. The cingulate cortex was detected for both groups, which has been reported to be essential for integrating inputs from various sources (including cognitive and emotional networks) and processing emotional information. Previous work has provided evidence that cingulate cortex undergoes a long period of development from infancy to late childhood (Bush, Luu, & Posner, 2000).

Most cortical regions detected in both age groups were located in frontal lobes. The frontal lobes are among the last cortex to mature anatomically and functionally, and may develop until mid‐twenties or even later (Rubia et al., 2000). Specially, the middle frontal gyrus identified on the left hemispheric prefrontal region has a stronger generic activation in the adults group compared with the adolescents. A significant increase in power with age has been reported for both left middle inferior frontal gyrus and right inferior frontal gyrus (Rubia et al., 2000). The inferior frontal cortex (both in left and right cerebrum) has also been reported to be activated during the emotional tasks for both adolescents and young adults (Iidaka et al., 2002). The superior frontal gyrus (SFG) is detected for both groups, which is thought to contribute to a variety of cognitive and motor functions. The lateral part of SFG plays an especially important role in the working memory network (Boisgueheneuc et al., 2006; Li et al., 2013). The middle frontal gyrus has been demonstrated to be related to memory retrieval and is observed in both groups, which is consistent with the studies illustrating the co‐activation of inferior and middle frontal gyrus for both young and older adults (Gunning‐Dixon et al., 2003; Rajah, Languay, & Grady, 2011). The right paracentral lobule is observed only in the young adult group, implying a stronger activation compared with the younger age, which provides additional evidence of the study by Langan (Langan & Seidler, 2011).

In the parietal lobe, increased functional specialization related to memory had been reported for inferior parietal cortex and this maturation happens during puberty (Gogtay et al., 2004; Rivera, Reiss, Eckert, & Menon, 2005). The identified postcentral gyrus is believed to be the first region to mature (Gogtay et al., 2004).

In the temporal lobe, the inferior and middle temporal gyrus were observed in the child group. According to a longitudinal study conducted by Gogtay (Gogtay et al., 2004), the inferior temporal lobe, similar to the maturation pattern with inferior frontal lobe, matures early and does not change much thereafter. The middle temporal gyrus is thought to be one of the heteromodal association sites involved in integration of memory, audiovisual association and object‐recognition functions (Gogtay et al., 2004). Moreover, the stronger extensive activation in dealing with the emotional task has been compared with the older group (Gunning‐Dixon et al., 2003), which is in accordance with our findings in the temporal lobe.

### 4.2 Major findings and contributions

We propose a novel framework to incorporate multi‐paradigm fMRI data to detect biomarkers related to brain maturation. From the most frequently identified brain regions, our work is consistent with the common knowledge about brain maturation during adolescence. Especially the abovementioned middle, inferior, and superior frontal gyrus, inferior parietal cortex, and inferior and middle temporal gyrus were identified to have similar activation pattern associations with age in previous work. We also found newly identified regions such as claustrum and insula, which have not previously been reported to be associated with development. These regions deserve further investigation on their roles in the emotion identification or working memory task.

Apart from the brain region detection, our proposed model also demonstrated its superior performance in other aspects. The higher concentration of energy distributed over the low frequency band of the learned graph Laplacian (Figure 4) demonstrated better extraction of well‐organized brain networks over the frequently used kernel based methods. This indicates that different paradigms provide complementary information for better characterization of brain networks based on an appropriately designed model. In addition, the robustness of our model was also evaluated and a high classification performance was achieved (Figure 3). Finally, both the high accuracy of classifying different age groups and subject consistency provided further evidence regarding the validity of our findings.

### 4.3 Limitations and future directions

One potential limitation is the sample size and scope of paradigms (284 subjects and two paradigms). Thus, a future direction is the testing of our proposed model on larger number of data sets from different modalities and paradigms, with further applications to mental disorder diagnosis and prognosis, and other clinical applications. Recent research also shows that the parcellation strategies can affect the constructed functional networks and the predictive power (de Reus & Van den Heuvel, 2013; Pervaiz, Vidaurre, Woolrich, & Smith, 2020). Therefore, another interesting direction would be to evaluate the stability of the proposed model with different parcellation atlases. Other concerns include the test–retest reliability of task fMRI (Elliott et al., 2020), and the reliability measured by intraclass correlation coefficient is poor compared with the resting state fMRI. Opposite result was observed in a recent study where predictive models built from task fMRI outperformed those from resting‐state fMRI in the classification of intelligence levels (Greene, Gao, Scheinost, & Constable, 2018). Because of this, the combination of multi‐paradigm fMRI studies including both resting state and tasks may increase the reliability. Recent work by Gao et al. (Gao, Greene, Constable, & Scheinost, 2019) demonstrated improved classification performance with the combination of resting state and task fMRI, and the reliability varies with different models and different data sets (the conclusion was based on the experiments on both PNC and Human Connectome Project [HCP] [van Essen et al., 2013] data set). We also conducted experiments based on resting state fMRI in our study and most of the identified regions were the same between two age groups and within the default mode network, which suggests that the DMN has similar patterns from childhood through puberty into adulthood. Thus resting‐state fMRI has limited contribution in identifying discriminative regions related to brain maturation and the classification performance of using resting‐state fMRI was worse compared with task fMRI. Therefore, we do not include the result of using resting‐state fMRI while believe the combination of multi‐paradigm fMRI is a more promising research direction. Finally, we recently propose a hypergraph learning based framework to capture higher order relationships in the brain network (Xiao et al., 2019). Accordingly, the proposed model can be expanded by the use of hypergraph Laplacian matrix, which can characterize more sophisticated structure of the brain.

## 5 CONCLUSION

In this paper, we proposed a multigraph Fourier analysis framework to study brain functional connectivity differences between children and young adults. Specifically, fMRI data were parcellated into regions of interest (ROI) according to a predefined functional atlas (Power et al., 2011). The two tasks of fMRI observations were treated as graph signals defined on the ROIs within the brain. The brain connectivity networks were jointly estimated based on the proposed model for both paradigms, followed by graph Fourier analysis with filtering to decompose the original signals into several frequency bands. Hub regions were detected in the low frequency domain based on the biological significance and energy concentration. In both synthetic and real data analysis, our proposed framework demonstrated the superiority over other GSP tools such as GFT, AGFT, and GLFT for the construction of brain connectivity network by considering the shared latent graph structures from both paradigms. From the analysis results of PNC data set, we identified the hub regions that were activated in child and young adult groups and explored their implication for brain maturation during puberty. We verified our findings with several existing studies and interpreted the significance of the detected regions.

## CONFLICT OF INTERESTS

The authors have no conflict of interest to disclose.

## Supporting information


**Appendix** S1: Supporting information

## Data Availability

All data for this study were collected from Philadelphia Neurodevelopmental Cohort (PNC). It is a large‐scale collaborative study between the Brain Behavior Laboratory at the University of Pennsylvania and the Center for Applied Genomics at the Children's Hospital of Philadelphia. The data are openly available at https://www.med.upenn.edu/bbl/philadelphianeurodevelopmentalcohort.html. All the source codes are written in Matlab including the public CVX toolbox (Version 2.1) for optimization and are available upon request to the authors.

## References

[hbm25410-bib-0001] Arain, M. , Haque, M. , Johal, L. , Mathur, P. , Nel, W. , Rais, A. , … Sharma, S. (2013). Maturation of the adolescent brain. Neuropsychiatric Disease and Treatment, 9, 449. 23579318 10.2147/NDT.S39776 PMC3621648

[hbm25410-bib-0002] Ashburner, J. , Barnes, G. , Chen, C. , Daunizeau, J. , Flandin, G. , Friston, K. , … Phillips, C. (2014). Spm12 manual. London, UK: Wellcome Trust Centre for Neuroimaging.

[hbm25410-bib-0003] Barabási, A.‐L. , & Albert, R. (1999). Emergence of scaling in random networks. Science, 286(5439), 509–512. 10521342 10.1126/science.286.5439.509

[hbm25410-bib-0004] Barch, D. M. , Burgess, G. C. , Harms, M. P. , Petersen, S. E. , Schlaggar, B. L. , Corbetta, M. , … van Essen, D. C. (2013). Function in the human connectome: Task‐fmri and individual differences in behavior. NeuroImage, 80, 169–189. 23684877 10.1016/j.neuroimage.2013.05.033 PMC4011498

[hbm25410-bib-0005] Bassett, D. S. , Bullmore, E. , Verchinski, B. A. , Mattay, V. S. , Weinberger, D. R. , & Meyer‐Lindenberg, A. (2008). Hierarchical organization of human cortical networks in health and schizophrenia. Journal of Neuroscience, 28(37), 9239–9248. 18784304 10.1523/JNEUROSCI.1929-08.2008 PMC2878961

[hbm25410-bib-0006] Belkin, M. , & Niyogi, P. (2003). Laplacian eigenmaps for dimensionality reduction and data representation. Neural Computation, 15(6), 1373–1396.

[hbm25410-bib-0007] Boisgueheneuc, F. d. , Levy, R. , Volle, E. , Seassau, M. , Duffau, H. , Kinkingnehun, S. , … Dubois, B. (2006). Functions of the left superior frontal gyrus in humans: A lesion study. Brain, 129(12), 3315–3328. 16984899 10.1093/brain/awl244

[hbm25410-bib-0008] Brown, T. T. , Lugar, H. M. , Coalson, R. S. , Miezin, F. M. , Petersen, S. E. , & Schlaggar, B. L. (2004). Developmental changes in human cerebral functional organization for word generation. Cerebral Cortex, 15(3), 275–290. 15297366 10.1093/cercor/bhh129

[hbm25410-bib-0009] Buckner, R. , Andrews‐Hanna, J. , & Schacter, D. (2008). The brains default network: Anatomy, function, and relevance to disease. Annals of the New York Academy of Sciences, 1124, 1–38. 18400922 10.1196/annals.1440.011

[hbm25410-bib-0010] Bush, G. , Luu, P. , & Posner, M. I. (2000). Cognitive and emotional influences in anterior cingulate cortex. Trends in Cognitive Sciences, 4(6), 215–222. 10827444 10.1016/s1364-6613(00)01483-2

[hbm25410-bib-0011] Chan, T. F. , Osher, S. , & Shen, J. (2001). The digital TV filter and nonlinear denoising. IEEE Transactions on Image Processing, 10(2), 231–241. 18249614 10.1109/83.902288

[hbm25410-bib-0012] Chung, F. R. (1997). Spectral graph theory. Number 92. Providence, RI: American Mathematical Society.

[hbm25410-bib-0013] de Reus, M. A. , & Van den Heuvel, M. P. (2013). The parcellation‐based connectome: Limitations and extensions. NeuroImage, 80, 397–404. 23558097 10.1016/j.neuroimage.2013.03.053

[hbm25410-bib-0014] Dong, X. , Thanou, D. , Frossard, P. , & Vandergheynst, P. (2016). Learning Laplacian matrix in smooth graph signal representations. IEEE Transactions on Signal Processing, 64(23), 6160–6173.

[hbm25410-bib-0015] Elliott, M. L. , Knodt, A. R. , Ireland, D. , Morris, M. L. , Poulton, R. , Ramrakha, S. , … Hariri, A. R. (2020). What is the test‐retest reliability of common task‐functional MRI measures? New empirical evidence and a meta‐analysis. Psychological Science, 31, 792–806. 32489141 10.1177/0956797620916786 PMC7370246

[hbm25410-bib-0016] Erdos, P. , & Rényi, A. (1960). On the evolution of random graphs. Publications of the Mathematical Institute of the Hungarian Academy of Sciences, 5(1), 17–60.

[hbm25410-bib-0017] Fair, D. A. , Cohen, A. L. , Power, J. D. , Dosenbach, N. U. , Church, J. A. , Miezin, F. M. , … Petersen, S. E. (2009). Functional brain networks develop from a local to distributed organization. PLoS Computational Biology, 5(5), e1000381. 19412534 10.1371/journal.pcbi.1000381 PMC2671306

[hbm25410-bib-0018] Gallese, V. , & Lakoff, G. (2005). The brain's concepts: The role of the sensory‐motor system in conceptual knowledge. Cognitive Neuropsychology, 22(3–4), 455–479. 21038261 10.1080/02643290442000310

[hbm25410-bib-0019] Gao, S. , Greene, A. S. , Constable, R. T. , & Scheinost, D. (2019). Combining multiple connectomes improves predictive modeling of phenotypic measures. NeuroImage, 201, 116038. 31336188 10.1016/j.neuroimage.2019.116038 PMC6765422

[hbm25410-bib-0020] Garrett, D. D. , Kovacevic, N. , McIntosh, A. R. , & Grady, C. L. (2012). The modulation of bold variability between cognitive states varies by age and processing speed. Cerebral Cortex, 23(3), 684–693. 22419679 10.1093/cercor/bhs055 PMC3823571

[hbm25410-bib-0021] Gogtay, N. , Giedd, J. N. , Lusk, L. , Hayashi, K. M. , Greenstein, D. , Vaituzis, A. C. , … Thompson, P. M. (2004). Dynamic mapping of human cortical development during childhood through early adulthood. Proceedings of the National Academy of Sciences of the United States of America, 101(21), 8174–8179. 15148381 10.1073/pnas.0402680101 PMC419576

[hbm25410-bib-0022] Grant, M. , Boyd, S. , and Ye, Y. (2015).“Cvx: Matlab software for disciplined convex programming (2008).”*Web page and software* Available from http://stanford.edu/boyd/cvx.

[hbm25410-bib-0023] Greene, A. S. , Gao, S. , Scheinost, D. , & Constable, R. T. (2018). Task‐induced brain state manipulation improves prediction of individual traits. Nature Communications, 9(1), 1–13. 10.1038/s41467-018-04920-3 PMC6052101 30022026

[hbm25410-bib-0024] Gunning‐Dixon, F. M. , Gur, R. C. , Perkins, A. C. , Schroeder, L. , Turner, T. , Turetsky, B. I. , … Gur, R. E. (2003). Age‐related differences in brain activation during emotional face processing. Neurobiology of Aging, 24(2), 285–295. 12498962 10.1016/s0197-4580(02)00099-4

[hbm25410-bib-0025] Holland, S. K. , Plante, E. , Byars, A. W. , Strawsburg, R. H. , Schmithorst, V. J. , & Ball, W. S., Jr. (2001). Normal fmri brain activation patterns in children performing a verb generation task. NeuroImage, 14(4), 837–843. 11554802 10.1006/nimg.2001.0875

[hbm25410-bib-0026] Hu, C. , Cheng, L. , Sepulcre, J. , El Fakhri, G. , Lu, Y. M. , and Li, Q. (2013). *Matched signal detection on graphs: Theory and application to brain network classification*. Paper presented at the International Conference on Information Processing in Medical Imaging, Springer. 1–12. 10.1007/978-3-642-38868-2_1 24683953

[hbm25410-bib-0027] Huang, W. , Goldsberry, L. , Wymbs, N. F. , Grafton, S. T. , Bassett, D. S. , & Ribeiro, A. (2016). Graph frequency analysis of brain signals. IEEE Journal of Selected Topics in Signal Processing, 10(7), 1189–1203. 28439325 10.1109/JSTSP.2016.2600859 PMC5400112

[hbm25410-bib-0028] Huettel, S. A. , Song, A. W. , & McCarthy, G. (2004). Functional magnetic resonance imaging (Vol. 1). MA: Sinauer Associates Sunderland.

[hbm25410-bib-0029] Iidaka, T. , Okada, T. , Murata, T. , Omori, M. , Kosaka, H. , Sadato, N. , & Yonekura, Y. (2002). Age‐related differences in the medial temporal lobe responses to emotional faces as revealed by fmri. Hippocampus, 12(3), 352–362. 12099486 10.1002/hipo.1113

[hbm25410-bib-0030] Jolles, D. D. , van Buchem, M. A. , Crone, E. A. , & Rombouts, S. A. (2010). A comprehensive study of whole‐brain functional connectivity in children and young adults. Cerebral Cortex, 21(2), 385–391. 20542991 10.1093/cercor/bhq104

[hbm25410-bib-0031] Kim, D. , Joung, J.‐G. , Sohn, K.‐A. , Shin, H. , Park, Y. R. , Ritchie, M. D. , & Kim, J. H. (2014). Knowledge boosting: A graph‐based integration approach with multi‐omics data and genomic knowledge for cancer clinical outcome prediction. Journal of the American Medical Informatics Association, 22(1), 109–120. 25002459 10.1136/amiajnl-2013-002481 PMC4433357

[hbm25410-bib-0032] Langan, J. , & Seidler, R. D. (2011). Age differences in spatial working memory contributions to visuomotor adaptation and transfer. Behavioural Brain Research, 225(1), 160–168. 21784106 10.1016/j.bbr.2011.07.014 PMC3170505

[hbm25410-bib-0033] Li, W. , Qin, W. , Liu, H. , Fan, L. , Wang, J. , Jiang, T. , & Yu, C. (2013). Subregions of the human superior frontal gyrus and their connections. NeuroImage, 78, 46–58. 23587692 10.1016/j.neuroimage.2013.04.011

[hbm25410-bib-0034] Li, Y. , Liu, J. , Gao, X. , Jie, B. , Kim, M. , Yap, P.‐T. , … Shen, D. (2019). Multimodal hyper‐connectivity of functional networks using functionally‐weighted lasso for mci classification. Medical Image Analysis, 52, 80–96. 30472348 10.1016/j.media.2018.11.006

[hbm25410-bib-0035] McHugh, M. L. (2013). The chi‐square test of independence. Biochemia medica: Biochemia Medica, 23(2), 143–149. 23894860 10.11613/BM.2013.018 PMC3900058

[hbm25410-bib-0036] Park, H.‐J. , & Friston, K. (2013). Structural and functional brain networks: From connections to cognition. Science, 342(6158), 1238411. 24179229 10.1126/science.1238411

[hbm25410-bib-0037] Passarotti, A. M. , Paul, B. M. , Bussiere, J. R. , Buxton, R. B. , Wong, E. C. , & Stiles, J. (2003). The development of face and location processing: An fmri study. Developmental Science, 6(1), 100–117.

[hbm25410-bib-0038] Pervaiz, U. , Vidaurre, D. , Woolrich, M. W. , & Smith, S. M. (2020). Optimising network modelling methods for fmri. NeuroImage, 211, 116604. 32062083 10.1016/j.neuroimage.2020.116604 PMC7086233

[hbm25410-bib-0039] Power, J. D. , Cohen, A. L. , Nelson, S. M. , Wig, G. S. , Barnes, K. A. , Church, J. A. , … Petersen, S. E. (2011). Functional network organization of the human brain. Neuron, 72(4), 665–678. 22099467 10.1016/j.neuron.2011.09.006 PMC3222858

[hbm25410-bib-0040] Rajah, M. N. , Languay, R. , & Grady, C. L. (2011). Age‐related changes in right middle frontal Gyrus volume correlate with altered episodic retrieval activity. Journal of Neuroscience, 31(49), 17941–17954. 22159109 10.1523/JNEUROSCI.1690-11.2011 PMC6634153

[hbm25410-bib-0041] Rivera, S. M. , Reiss, A. , Eckert, M. A. , & Menon, V. (2005). Developmental changes in mental arithmetic: Evidence for increased functional specialization in the left inferior parietal cortex. Cerebral Cortex, 15(11), 1779–1790. 15716474 10.1093/cercor/bhi055

[hbm25410-bib-0042] Rubia, K. , Overmeyer, S. , Taylor, E. , Brammer, M. , Williams, S. , Simmons, A. , … Bullmore, E. (2000). Functional frontalisation with age: Mapping neurodevelopmental trajectories with fMRI. Neuroscience & Biobehavioral Reviews, 24(1), 13–19. 10654655 10.1016/s0149-7634(99)00055-x

[hbm25410-bib-0043] Sandryhaila, A. , & Moura, J. M. (2013). Discrete signal processing on graphs. IEEE Transactions on Signal Processing, 61(7), 1644–1656.

[hbm25410-bib-0044] Sandryhaila, A. , & Moura, J. M. (2014). Big data analysis with signal processing on graphs: Representation and processing of massive data sets with irregular structure. IEEE Signal Processing Magazine, 31(5), 80–90.

[hbm25410-bib-0045] Satterthwaite, T. D. , Connolly, J. J. , Ruparel, K. , Calkins, M. E. , Jackson, C. , Elliott, M. A. , … Gur, R. E. (2016). The Philadelphia neurodevelopmental cohort: A publicly available resource for the study of normal and abnormal brain development in youth. NeuroImage, 124, 1115–1119. 25840117 10.1016/j.neuroimage.2015.03.056 PMC4591095

[hbm25410-bib-0046] Satterthwaite, T. D. , Elliott, M. A. , Ruparel, K. , Loughead, J. , Prabhakaran, K. , Calkins, M. E. , … Gur, R. E. (2014). Neuroimaging of the Philadelphia neurodevelopmental cohort. NeuroImage, 86, 544–553. 23921101 10.1016/j.neuroimage.2013.07.064 PMC3947233

[hbm25410-bib-0047] Schlaggar, B. L. , Brown, T. T. , Lugar, H. M. , Visscher, K. M. , Miezin, F. M. , & Petersen, S. E. (2002). Functional neuroanatomical differences between adults and school‐age children in the processing of single words. Science, 296(5572), 1476–1479. 12029136 10.1126/science.1069464

[hbm25410-bib-0048] Schölkopf, B. , & Smola, A. J. (2002). Learning with kernels: Support vector machines, regularization, optimization, and beyond. Cambridge, MA: MIT press.

[hbm25410-bib-0049] Shuman, D. I. , Narang, S. K. , Frossard, P. , Ortega, A. , & Vandergheynst, P. (2013). The emerging field of signal processing on graphs: Extending high‐dimensional data analysis to networks and other irregular domains. IEEE Signal Processing Magazine, 30(3), 83–98.

[hbm25410-bib-0050] Sporns, O. (2010). Networks of the brain. Cambridge, MA: MIT press.

[hbm25410-bib-0051] Sundermann, B. , Herr, D. , Schwindt, W. , & Pfleiderer, B. (2014). Multivariate classification of blood oxygen level‐dependent fMRI data with diagnostic intention: A clinical perspective. American Journal of Neuroradiology, 35(5), 848–855. 24029388 10.3174/ajnr.A3713 PMC7964550

[hbm25410-bib-0052] Tsuda, K. , Shin, H. , & Schölkopf, B. (2005). Fast protein classification with multiple networks. Bioinformatics, 21(suppl_2), ii59–ii65. 16204126 10.1093/bioinformatics/bti1110

[hbm25410-bib-0053] van Essen, D. C. , Smith, S. M. , Barch, D. M. , Behrens, T. E. , Yacoub, E. , & Ugurbil, K. (2013). The wu‐minn human connectome project: An overview. NeuroImage, 80, 62–79. 23684880 10.1016/j.neuroimage.2013.05.041 PMC3724347

[hbm25410-bib-0054] Wang, J. , Xiao, L. , Wilson, T. W. , Stephen, J. M. , Calhoun, V. D. , & Wang, Y.‐P. (2020). Examining brain maturation during adolescence using graph laplacian learning based fourier transform. Journal of Neuroscience Methods, 338, 108649. 32165231 10.1016/j.jneumeth.2020.108649 PMC7936225

[hbm25410-bib-0055] Wee, C.‐Y. , Yap, P.‐T. , Zhang, D. , Wang, L. , & Shen, D. (2014). Group‐constrained sparse fMRI connectivity modeling for mild cognitive impairment identification. Brain Structure and Function, 219(2), 641–656. 23468090 10.1007/s00429-013-0524-8 PMC3710527

[hbm25410-bib-0056] Xia, M. , Wang, J. , & He, Y. (2013). Brainnet viewer: A network visualization tool for human brain connectomics. PLoS One, 8(7), e68910. 23861951 10.1371/journal.pone.0068910 PMC3701683

[hbm25410-bib-0057] Xiao, L. , Wang, J. , Kassani, P. H. , Zhang, Y. , Bai, Y. , Stephen, J. M. , … Wang, Y.‐P. (2019). Multi‐hypergraph learning based brain functional connectivity analysis in fmri data. IEEE Transactions on Medical Imaging, 39, 1746–1758. 31796393 10.1109/TMI.2019.2957097 PMC7376954

