# Supplementary material for: Functional network estimation using multigraph learning with application to brain maturation study
Source: Hum Brain Mapp. 2021 Mar 31;42(9):2880–92. doi: 10.1002/hbm.25410 (PMC8127152; doi:10.1002/hbm.25410)
Supplement: Supplementary file 1 — Appendix S1: Supporting information [file HBM-42-2880-s001.pdf]

Supplementary file

Junqi Wang[1]**, Li Xiao**[1]**, Wenxing Hu**[1]**, Gang Qu**[1]**, Tony W. Wilson**[2]**, Julia M. Stephen**[3]**, Vince D. Calhoun**[4]**, Yu-Ping Wang**[1,*]
[1]Department of Biomedical Engineering, Tulane University, New Orleans, LA, USA
[2]Department of Neurological Sciences, University of Nebraska Medical Center,Omaha, NE, USA
[3]Mind Research Network, Albuquerque, NM, USA
[4]Tri-institutional center for Translational Research in Neuroimaging and Data Science (TReNDS) {Georgia State University, Georgia Institute of Technology, Emory University}, Atlanta, GA

1 The optimization involved in GLFT (section2.2.2)

The smooth signal Y and the graph Laplacian were estimated using the following objective function:

$$\begin{aligned} \min_{L\in\mathbb{R}^{n\times n}, Y\in\mathbb{R}^{n\times p}} & \alpha\|X-Y\|_F^2 + tr(Y^T LY) + \beta\|L\|_F^2 \\ s.t\ & tr(L)=n, \\ & L_{ij}=L_{ji}\leq 0, (i\neq j), \\ & L\cdot\mathbb{1}=\mathbb{0}, \end{aligned} \tag{1}$$

where α, β are two positive regulators making the tradeoff of the fitting fidelity and penalty term. The second term in the objective function modulates the total variation and the third one (Frobenius norm of L) is the penalty term to control the off-diagonal elements in L. The constraints of the objective function ensure the learned graph Laplacian is non-trivial and valid.

2 The optimization problem involved in Multi-graph Learning (section2.3.1)

Estimation of the graph Laplacian for single modality:

$$\begin{aligned} \min_{L^{(k)}} & tr(X^{(k)^T} L^{(k)} X^{(k)}) + \beta\|L^{(k)}\|_F^2 \\ s.t\ & tr(L^{(k)})=n, \\ & L_{ij}^{(k)}=L_{ji}^{(k)}\leq 0, (i\neq j), \\ & L^{(k)}\cdot\mathbb{1}=\mathbb{0}. \end{aligned} \tag{2}$$

3 The graph filters designed for Graph Fourier transform (section2.3.3)

Specifically for $k-th$ paradigm, if we sort the eigenvalues of its graph Laplacian in an increasing order, $\lambda_l^{(k)}$ and $\lambda_m^{(k)}$ can be adopted as the thresholds to separate low and high frequencies.

$$\begin{aligned} \Lambda^{(k)} =& diag(\lambda_1^{(k)}, \lambda_2^{(k)}, ..., \lambda_l^{(k)}, 0, ..., 0) + \\ & diag(0, ..., 0, \lambda_{l+1}^{(k)}, ..., \lambda_m^{(k)}, 0, ..., 0) + \\ & diag(0, ..., 0, \lambda_{m+1}^{(k)}, ..., \lambda_n^{(k)}). \end{aligned} \tag{3}$$

The corresponding graph filter for each frequency range can be defined as:

$$\mathscr{G}_{low} = diag(\underbrace{1, 1, ..., 1}_{l}, 0, ..., 0) \tag{4}$$

$$\mathscr{G}_{mid} = diag(\underbrace{0, ..., 0}_{l}, \underbrace{1, ..., 1}_{m-l}, 0, ..., 0) \tag{5}$$

$$\mathscr{G}_{high} = diag(0, ..., 0, \underbrace{1, ..., 1}_{n-m}). \tag{6}$$
